# SYNTAXIN OF PLANTS132 Regulates Root Meristem Activity and Stem Cell Niche Maintenance via RGF-PLT Pathways

**DOI:** 10.3390/ijms26052123

**Published:** 2025-02-27

**Authors:** Mingjing Wang, Fumeng He, Wei Zhang, Chong Du, Linlin Wang, Jia Sui, Fenglan Li

**Affiliations:** 1 College of Life Sciences, Northeast Agricultural University, Harbin 150030, China; wangmingjing2021@163.com (M.W.);; 2 Key Laboratory of Soybean Molecular Design Breeding, State Key Laboratory of Black Soils Conservation and Utilization, Northeast Institute of Geography and Agroecology, Chinese Academy of Sciences, Changchun 130102, China

**Keywords:** *SYNTAXIN OF PLANTS132* (*SYP132*), auxin-PLT pathway, endosome-PM cycling, RGF-PLT pathway, root stem cell

## Abstract

Root growth and development are contingent upon continuous cell division and differentiation in root tips. In this study, we found that the knockdown of the syntaxin gene *SYNTAXIN OF PLANTS132* (*SYP132*) in *Arabidopsis thaliana* resulted in a significant reduction in root meristem activity and disruption of root stem cell niche (SCN) identity. The *SYP132* knockdown mutant exhibits a compromised SCN characterized by an increased number of quiescent center (QC) cells, abnormal columella stem cells (CSCs), reduced meristem size, and subsequent inhibition of root growth. In *syp132*, vesicle transport of PIN proteins is disrupted, leading to altered auxin distribution and decreased expression of the auxin-response transcription factors PLETHORA 1 (*PLT1*) and PLETHORA 2 (*PLT2*). Furthermore, the transcription level of the precursor of *root meristem growth factor 1* (*RGF1*) is also modified in *syp132*. The reduction in *PLT2* transcription and protein levels along with defects in the root SCN are partially rescued by the application of synthesized RGF1. This finding suggests that both the auxin-PLT and RGF-PLT pathways are interconnected through SYP132-mediated vesicle transport. Collectively, our findings indicate that SYP132 regulates the PLT pathway to maintain the root stem cell niche (SCN) in an RGF1-dependent manner.

## 1. Introduction

Plant roots anchor the plants in the soil, facilitating the acquisition of water and nutrients, the synthesis of plant hormones, and storage functions [1]. The development of root systems involves various unique strategies to adapt to changing soil and environmental conditions. Root structure is established and maintained through cell division and differentiation. In the root apical meristem (RAM), a small group of undifferentiated initial or stem cells surround the quiescent center (QC) and have the potential to divide. These cells maintain the undifferentiated state of stem cells through short-range, non-autonomous signaling [2]. The QC and surrounding stem cells constitute the stem cell niche (SCN) [3].

PLETHORA (PLT) proteins are AP2 domain transcription factors (TFs). During embryogenesis, they are primarily expressed in the QC and provascular tissues, where their expression domains overlap with the maximum distribution of auxin. Subsequently, PLTs function downstream of auxin to regulate the maintenance of the root SCN [4,5]. PLTs regulate root development in a dose-dependent manner: higher levels of PLT expression promote stem cell identity and maintenance, while lower levels influence cell division and differentiation within the transition zones [2,6]. It was found that, in the *plt1-4 plt2-2* double mutant, the QC identity is lost, whereas PLT2 overexpression results in a substantial increase in proximal meristem cell number in roots [6]. Additionally, peptide hormones from the root growth factor (RGF) family are essential for SCN maintenance by regulating the expression of PLTs [6,7]. RGF gene multiple mutants exhibit a decreased number of meristematic cells in the RAM, which can be rescued by synthesized RGF peptides. Mature RGFs are tyrosine-sulfated peptides that require a tyrosylprotein sulfotransferase (TPST) to add sulfate to their tyrosine residues [8,9]. A *TPST1/AQC1* gene-deficient mutant exhibited a defective SCN, which could be rescued by RGFs. Recently, RGF receptors (RGFRs) were identified that bind RGFs and facilitate PLT gradient formation in the RAM to regulate root meristem development [10]. RGFs belong to secreted peptides, which are important signals for intercellular communication and determining the identity of neighboring cells [10,11,12]. However, the mechanisms underlying how these peptides are secreted remain so far elusive. In addition to *PLT* genes, GRAS family TFs, SHR, and SCR also regulate SCN maintenance in parallel with the PLT pathway [13,14,15,16]. SHR and SCR are required for the asymmetric division of stem cell daughter cells for the formation of the cortex and the endodermis [17].

A SNARE (soluble N-ethylmaleimide-sensitive factor attachment protein receptor) complex is a machinery for guiding membrane fusion between the vesicles and the target membrane in vesicle trafficking [18]. According to their localization, SNAREs are divided into v-SNAREs (vesicle SNAREs) and t-SNAREs (target membrane SNAREs) [19]. Usually, t-SNAREs form a SNARE complex with their partner v-SNARE to mediate membrane trafficking. In *Arabidopsis thaliana*, there are 64 SNARE gene-encoding proteins that are broadly distributed on various organelles, such as the endoplasmic reticulum (ER), Golgi apparatus, endosome, vacuole, cytosol, plasma membrane (PM), etc. [20,21]. The functions of some plant SNAREs have been revealed. They are involved in diverse processes, such as cytokinesis [22,23], abscisic acid signaling [24], shoot gravitropism [25,26], osmotic stress [27,28], infectious pathogens resistance [29], and bacterial resistance [30].

PM-localized t-SNAREs have been suggested to allow for the fusion of different types of vesicles to the PM in response to specific development and biotic/abiotic cues. For example, SYP121 is involved in the regulation of K^+^ channels and resistance against barley powdery mildew by positively regulating the formation of cell wall appositions (papillae) at fungal entry sites [31,32]. In addition, SYP121 plays a key role in the proper delivery of PM intrinsic protein 2;7 (PIP2;7) to the PM, mediating passive movement of water and small neutral solutes through the biological membranes [33]. The *syp121* single mutant did not show any defects in non-host resistance, but growth defects were shown in the *syp121 syp122* double mutant, suggesting SYP121 and SYP122 might have overlapping functions in plant growth and development [34]. KNOLLE (SYP111) is specifically expressed in the M-phase of the cell cycle, targeting to the plane of cell division and degrading in the vacuole at the end of cytokinesis [35]. SYP132 could recover the *knolle* phenotype, whereas the modified KNOLLE replaced with the SYP132 SNARE domain could recover the *knolle* phenotype. However, SYP121 driven by its native or KNOLLE promoter could not rescue *knolle* [36]. These results demonstrate that PM-localized SYP132 might have an overlapping function with KNOLLE, whereas SYP121 and KNOLLE might have distinct roles, perhaps by forming highly specialized complexes. Recently, Ichikawa et al. demonstrated that SYP132 and SYP123 act in a coordinated fashion to mediate tip-focused membrane trafficking for root hair tip growth [37]. As a plasma membrane-localized t-SNARE, *SYP132* plays multiple critical roles in plant biology. It exhibits dual localization to both the plasma membrane and the Golgi apparatus, indicating its involvement in secretion and endocytosis [38]. SYP132 is essential for cytokinesis and the maturation of the cell plate during plant cell division [39]. Research has demonstrated that SYP132 interacts with other SNARE proteins, such as KNOLLE, to facilitate vesicle fusion in cell plate formation. Additionally, SYP132 enhances plant tolerance to drought and salinity stress by regulating ion transport and stomatal closure [40,41]. In legumes, SYP132 is vital for rhizobial infection and the formation of symbiosomes during root nodule development [42,43]. Despite these insights, the mechanisms underlying SYP132-regulated membrane trafficking remain poorly understood.

In this study, we demonstrate that the plasma membrane-localized *SYP132* regulates polar auxin transport (PAT) by modulating the endosome-plasma membrane trafficking of PIN proteins in collaboration with the v-SNAREs *VAMP721* and *VAMP722*. The defects observed in the *syp132-1* mutant indicate a significant role for *SYP132* in maintaining the SCN and promoting root development. Furthermore, our findings reveal that the regulation of *SYP132* in maintaining the root stem niche is dependent on the RGF and PLT pathways.

## 2. Results

### 2.1. SYP132 Plays a Crucial Role in Both Root Growth and Development

**Figure 1:**
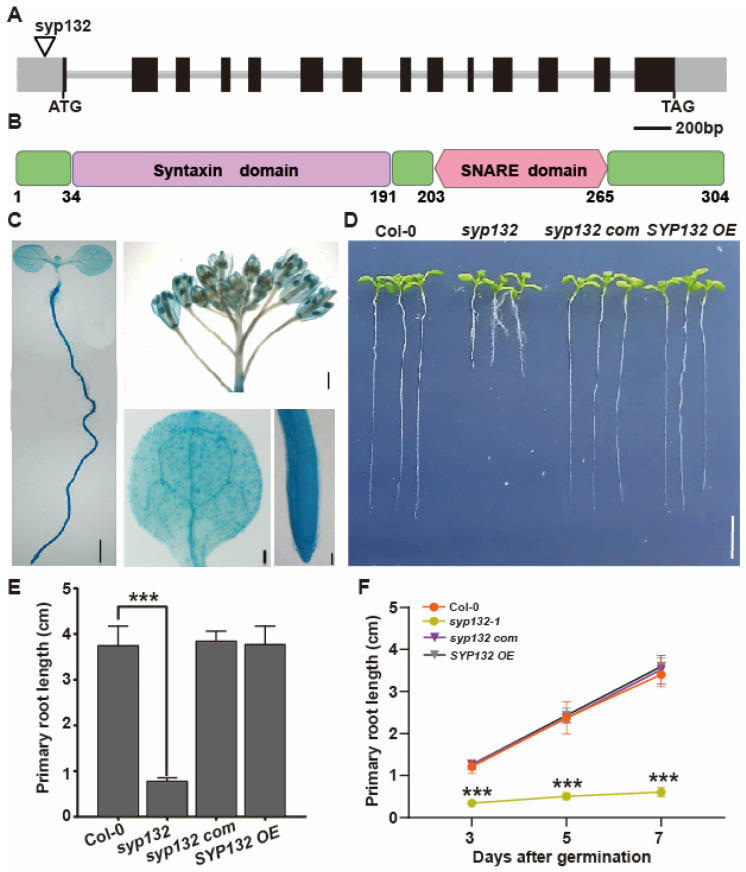
*SYP132* plays an essential role in root growth and development. (**A**,**B**) Schematic structure of SYP132 gene (**A**) and protein (**B**). Dark gray boxes represent exons, solid lines represent introns, and light gray boxes represent UTRs, respectively. T-DNA insertion site of *syp132* is indicated by an unfilled triangle. SYP132 is predicted to be a 34.2 kDa protein that contains one syntaxin domain and one SNARE domain. The syntaxin domains are represented by purple boxes, while the SNARE domains are indicated in pink. (**C**) GUS staining of 7-day-old *SYP132pro:GUS* transgenic seedlings. Scale bars: 0.5 cm. (**D**) Phenotypes of 7-day-old seedlings of the indicated genotypes. Scale bars: 1 cm. (**E**) Statistics of primary root length of 7-day-old seedlings shown representatively in (**D**) Values are means ± SD (*n* ≥ 60) from three biological replications. (**F**) Statistics of primary root growth of seedlings of the indicated genotypes. Values are means ± SD (*n* ≥ 60) from three biological replications. *** *p* < 0.001; Student’s *t*-test.

SYP132 is a member of the SYP13 subgroup within the SYP1 family. The SYP132 gene comprises 13 exons and 12 introns (Figure 1A). This gene is predicted to encode a protein of 34.2 kDa that contains one syntaxin domain and one SNARE domain (Figure 1B). To investigate the tissue expression profile, transgenic plants expressing the *SYP132pro:GUS* construct were generated. SYP132 expression was observed in all tissues, consistent with the findings from a previous study [44]. GUS signals were detected in the cotyledon, root, and floral structures, including the stigma, stamen, petal, and sepal (Figure 1C). The *SYP132* promoter exhibited high expression activity uniformly throughout the entire root (Figure 1C), suggesting that *SYP132* plays a critical role in root development and growth. Genotyping confirmed T-DNA insertion into the *SYP132* mutant, which was obtained from NASC (The Nottingham Arabidopsis Stock Centre). The analysis revealed that the inserted T-DNA was located in the 157 bp region of the 5′-UTR upstream of *SYP132*, thereby identifying it as a *syp132* mutant (Figure 1A and Figure S1). Complementation of the *syp132* allele with a construct containing the *SYP132* promoter sequence driving the *SYP132* cDNA (*syp132* com line) effectively rescued the diminished expression of *SYP132* in the *syp132* mutant (Figure S1). In contrast, the *p35S::TAP-SYP132* construct resulted in significantly elevated expression of *SYP132* in a wild-type background (*SYP132* OE lines) (Figure S1).

Seven-day-old *syp132* seedlings exhibit significantly shorter primary roots (PR), indicating delayed root growth and development (Figure 1D–F). The PR length of these seedlings is approximately 20% that of wild-type (Col-0) seedlings (Figure 1E,F). These observed defects underscore the crucial role of *SYP132* in root development and overall plant growth. Additionally, eight-week-old *syp132* mutant plants display dwarfism, characterized by small and empty siliques (Figure S2), suggesting that *SYP132* is also involved in embryogenesis. To maintain homozygous *syp132* plants, we employed *syp132-1^+^/^−^* heterozygous seeds. To ascertain whether *SYP132* deficiency is responsible for these phenotypes, we performed complementation of the *syp132* mutant by introducing a native promoter-driven *SYP132* genomic fragment (*SYP132pro:gSYP132*) into *syp132^+^/^−^* heterozygous plants. The complemented homozygous *syp132* plants exhibited a recovery of all phenotypes (Figure 1D–F), supporting the hypothesis that the knockdown of *SYP132* is the underlying cause of the observed *syp132* phenotypes.

### 2.2. Knockdown Line of SYP132 Shows Defects on RAM and SCN Maintenance

**Figure 2:**
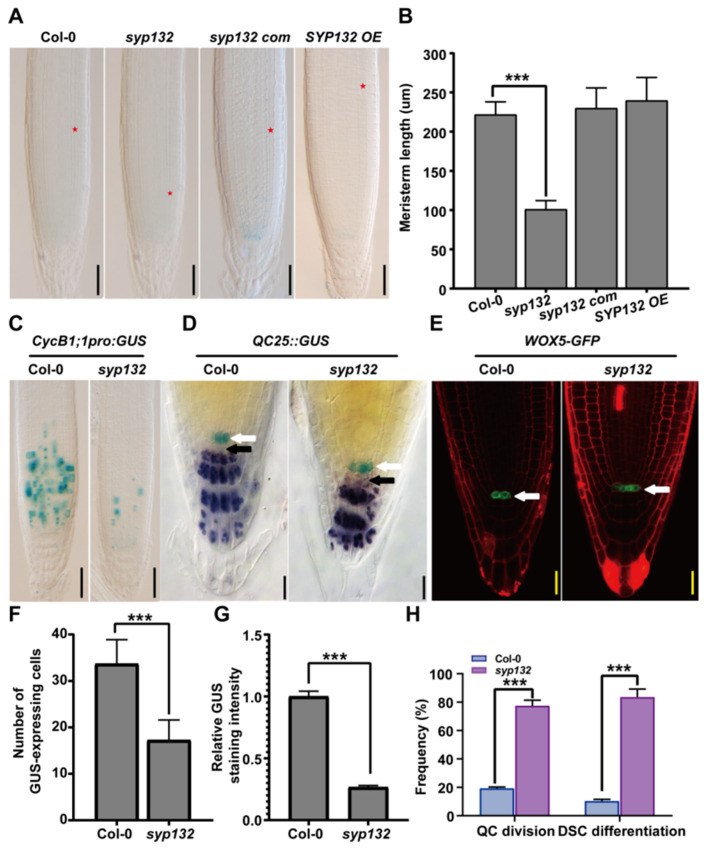
*SYP132* exhibits extra QC cells and defective CSC maintenance. (**A**) Nomarski images of PR from 7-day-old seedlings. Red asterisk indicates cortex transition zones. Bars, 50 µm. (**B**) Statistics of RAM length shown representatively in (**A**). Values are means ± SD (*n* ≥ 50) from three biological replications. *** *p* < 0.001; Student’s *t*-test. (**C**) Expression of *CycB1;1pro:GUS* in PR from 7-day-old seedlings. Bars, 50 μm. (**D**) Nomarski images of *QC25::GUS*-expressing cells and starch granules stained by Lugol’s solution in roots of seven-day-old Col-0 and *syp132* seedlings. White arrow: indicates QC cells. Black arrow: indicates CSCs. Bars, 50 µm. (**E**) Expression of *WOX5-GFP*. White arrow: indicates QC cells. Bars, 50 µm. (**F**,**G**) Statistics of relative *CycB1;1::GUS* staining intensities determined by Image software (ImageJ, Version: 1.53t) (**G**) and GUS-expressing cell number in RAM (**F**) shown representatively in (**C**) Values are means ± SD (*n* ≥ 50) from three biological replications. (**H**) Statistics of frequencies of QC cell division and CSC differentiation in roots of *syp132*. Values are means ± SD (*n* ≥ 100) from three biological replications. *** *p* < 0.001; Student’s *t*-test.

Seven-day-old syp132 seedlings exhibited root growth inhibition. The RAM size in *syp132* was significantly decreased compared to that in Col-0 seedlings (Figure 2A,B). A continuous decline in root growth rates was subsequently observed in the *syp132* mutant (Figure 1F). Consistent with this observation, *syp132* mutant seedlings displayed a reduced RAM size due to a decrease in cell number (Figure 2A,B). To investigate changes in meristematic activity, the *CYCB1;1pro:GUS* construct, a cell-cycle G2/M marker, was introduced into *syp132*. This resulted in the decreased expression of *CYCB1;1pro:GUS* in *syp132* (Figure 2C,F,G), suggesting that the reduced RAM size in the *syp132* mutant is associated with diminished mitotic activity.

The root SCN is composed of several mitotically inactive QC cells and their surrounding stem cells, providing a source of cells for RAM [45]. The quiescent center (QC) cells were monitored using two QC-specific markers: *QC25pro:GUS* and *WOX5pro:GFP*. It was observed that 64% of *syp132* roots contain three or more QC cells, significantly higher than the 31% observed in Col-0 roots (Figure 2D,E,H), suggesting increased mitotic activity in the QC of the *syp132* mutant (Figure 2D,E, white arrows). Lugol staining revealed that columella stem cells (CSCs) in Col-0 do not contain starch granules in contrast to the adjacent differentiated CSCs. However, in *syp132*, CSCs were found to contain starch granules (Figure 2D, black arrows), indicating that CSCs in the root tips of *syp132* have undergone differentiation. Collectively, these findings suggest that the maintenance of the RAM and CSCs is disrupted in the *syp132* mutant.

### 2.3. SYP132 Affects Auxin Response via Regulating PIN1 Intracellular Trafficking

**Figure 3:**
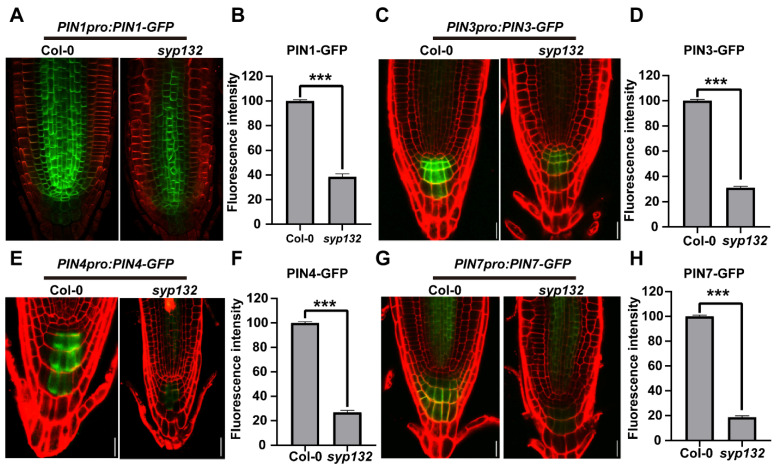
Expression of auxin reporter genes and auxin efflux carriers. Alteration of PIN1, PIN3, PIN4, and PIN7 expression in *syp132*. Expression of *PIN1pro::PIN1-GFP* (**A**,**B**), *PIN2pro::PIN2-GFP* (**C**,**D**), *PIN3pro::PIN3-GFP* (**E**,**F**), and *PIN7pro::PIN7-GFP* (**G**,**H**) in root tips of seven-day-old Col-0 and *syp132.* Bars, 20 µm. Values are means ± SD (*n* ≥ 50) from three biological replications. *** *p* < 0.001; Student’s *t*-test.

Auxin plays a crucial role in root development and SCN maintenance [46]. The defects observed in the RAM and SCN of the *syp132* mutant suggest that auxin signaling is compromised [47,48,49]. Auxin transport is mediated by PIN transporters. To investigate this, we crossed several marker lines, including *PIN1pro:PIN1-GFP*, *PIN3pro:PIN3-GFP*, *PIN4pro:PIN4-GFP*, and *PIN7pro:PIN7-GFP*, with *PIN4pro:GUS* and *PIN7pro:GUS* reporter lines into the *syp132^+/−^* heterozygous line. As anticipated, the signals for PIN3-GFP, PIN4-GFP, and PIN7-GFP were significantly reduced in the columella cells of *syp132* (Figure 3A–H). Additionally, lower expression levels of *PIN4pro:GUS* and *PIN7pro:GUS* were evident in the roots of *syp132* (Figure S3). We conducted a further analysis of the expression levels of *PIN1, PIN3, PIN4*, and *PIN7* in the roots of *syp132* by employing RT-qPCR. As anticipated, the expression levels of *PIN1, PIN3, PIN4*, and *PIN7* were significantly reduced in the roots of *syp132* (Figure S4).

**Figure 4:**
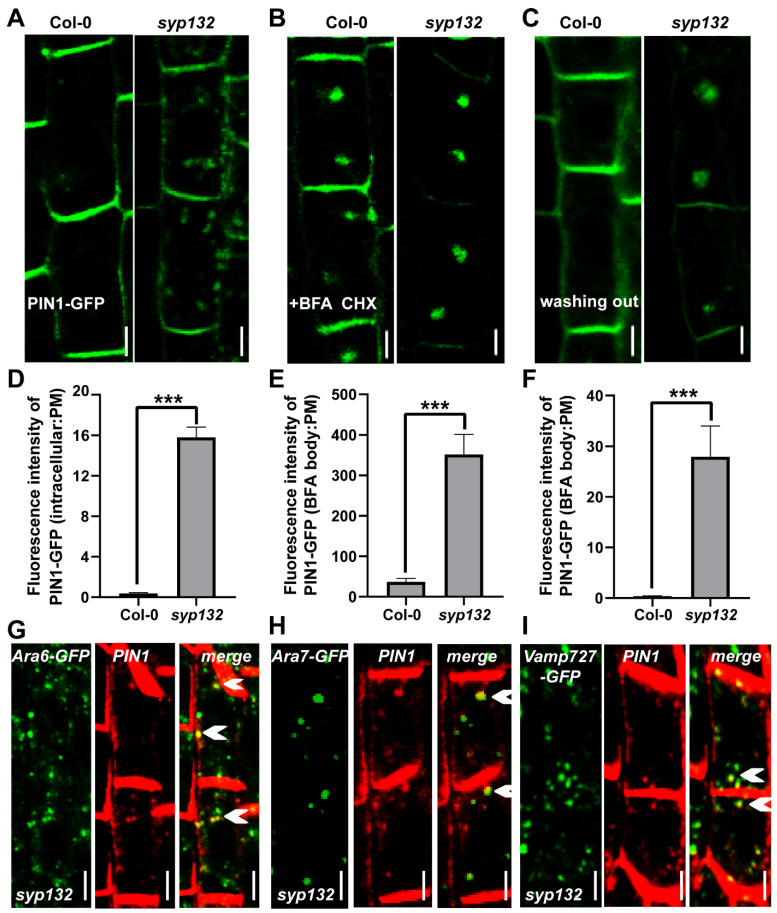
*SYP132* is defective on PIN1 targeting to the PM. (**A**) PIN1-GFP subcellular localization in Col-0 and *syp132*. Bars, 2 µm. (**B**) Application of 50 µM BFA in presence of 50 µM CHX for 45 min to PR from 6-day-old seedlings. Bars, 2 µm. (**C**) 1.5 h treatment with 50 μM BFA and 50 µM CHX, followed by a 1.5 h washing out with half MS liquid medium. Bars, 2 µm. (**D**) Ratio of PIN1-GFP fluorescence in aggregates to that on PM in (**A**). *n* = 36 cells. (**E**,**F**) Ratio of PIN1-GFP fluorescence in BFA bodies to that on PM in (**B**,**C**). *n* = 52 cells. ***, *p* < 0.001. (**G**–**I**) Immunolocalization of PIN1 (red) with Ara6-GFP, Ara7-GFP, and VAMP727-GFP (green) in *syp132*, respectively. (**G**) White arrows indicate ectopic endogenous PIN1 that is colocalized with ARA6-GFP in *syp132*. (**H**) White arrows indicate ectopic endogenous PIN1 that is colocalized with ARA7-GFP in *syp132*. (**I**) White arrows indicate ectopic endogenous PIN1 that is colocalized with Vamp727-GFP in *syp132*. Bars, 2 µm.

These findings indicate that the expression of PINs is affected in *syp132*. Further analysis focused on the subcellular localization of PIN1-GFP in *syp132*. In Col-0 plants, PIN1-GFP is predominantly localized on the basal side of the plasma membrane (PM) in stele cells (Figure S5). In contrast, 58% of root stele cells in *syp132* exhibited intracellular accumulation of PIN1-GFP alongside its basal localization (Figure 4A). The fluorescence intensity of intracellular PIN1-GFP was 15.8% of that observed on the PM, representing an 89.5-fold increase compared to Col-0 (0.0017%) (Figure 4D). The intracellular trafficking of PIN1 involves both endocytosis and exocytosis, with its polar localization dependent on BFA-sensitive intracellular transport [50,51]. To gain further insight into the essence of PIN1 intracellular trafficking, we applied BFA, a reversible inhibitor that blocks exocytosis in the presence of a de novo protein synthesis inhibitor cycloheximide (CHX) [50,52,53]. It was observed that BFA-induced PIN1-GFP intracellular aggregation in *syp132* was enhanced, and PM-localized PIN1-GFP was depleted (Figure 4B). The fluorescence intensity in BFA bodies to that on the PM in *syp132* was 8.8 times higher than that in Col-0, suggesting that the PIN1 trafficking mediated by SYP132 is super BFA sensitive (Figure 4E). To determine which direction of PIN1 trafficking, anterograde or retrograde transport, is defective in *syp132*, we performed a BFA washing-out experiment. After a 90 min treatment of 50 μM BFA with 50 μM CHX, followed by a 90 min washing out with 1/2 MS liquid medium, PIN1-GFP was completely recovered to the PM in Col-0. However, a mass of BFA-induced PIN1-GFP aggregates remained in *syp132* (Figure 4C). The fluorescent intensity in BFA compartments to the PM in *syp132* was 75.5 times higher than that in Col-0 (Figure 4F). These results indicate that anterograde transport of PIN1 from the endosome to the PM is defective in *syp132*, namely, SYP132 is required for PIN1 targeting to the PM. To identify the exact localization of the ectopic PIN1 in *syp132*, we crossed the early endosome markers *CaMV35S_pro_:VAMP727-GFP* and *CaMV35S_pro_:ARA7-GFP* [21] and late endosome marker *CaMV35S_pro_:ARA6-GFP* [54] with the *syp132*^+/−^ heterozygous line, respectively. Then, we performed immunolocalization with anti-PIN1 and anti-GFP antibodies using the mentioned crossing lines. The results showed co-localization of ectopic endogenous PIN1 with ARA6-GFP, ARA7-GFP, and VAMP727-GFP (Figure 4G–I, white triangles), indicating that PIN1 is accumulated in early and later endosomes in *syp132*. All the data suggest that knockdown of SYP132 resulted in defects on constitutive endocytic cycling, which led to ectopic and lower accumulation of auxin transporters; as a consequence, auxin distribution was disturbed.

### 2.4. SYP132 Cooperates with VAMP721/VAMP722 to Regulate PIN1 Intracellular Trafficking

**Figure 5:**
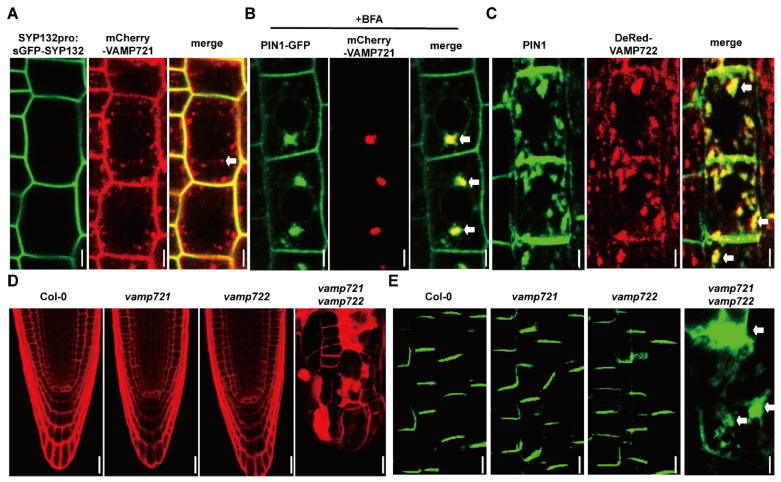
Co-localization of SYP132, PIN1, and VAMP721/722. (**A**) sGFP-gSYP132 co-localized (green) with mCherry-VAMP721 (red) on the PM. White arrows represent that mCherry-VAMP721 is also localized on an intracellular compartment. Bars, 5 µm. (**B**) Co-localization of PIN1-GFP (green) with mCherry-VAMP721 (red) at BFA compartments (yellow). White arrows indicate that, following BFA treatment, mCherry-VAMP721 was translocated to the BFA compartment and colocalized with PIN1-GFP. Bars, 5 µm. (**C**) Immunolocalization of PIN1 (green) with DsRed:VAMP722 (red) in *syp132*. White arrows indicate the colocalization of endogenous PIN1 and DsRed-VAMP722 in intracellular aggregates within *syp132*. Bars, 2 µm. (**D**) mPS-PI staining of PR from *vamp721 vamp722* double mutant. Bars, 5 µm. (**E**) PIN1-GFP in *vamp721 vamp722* double mutant. Bars, 5 µm.

It is reported that VAMP721 and VAMP722 are PM-destined SNAREs and play critical roles in plant growth and development [21,44,55]. Since VAMP721 and VAMP722 physically interacted with SYP132 in vitro and in vivo [56], we investigated their role on PIN1 trafficking. We crossed *VAMP721pro:mCherry-VAMP721* with the *SYP132pro:sGFP-gSYP132* and *PIN1pro:PIN1-GFP* expressing lines, and *VAMP722pro:DsRed-gVAMP722* with the syp132-1^+/−^ heterozygous line. In Col-0, *SYP132pro:sGFP-SYP132* co-localized with mCherry-VAMP721 at the plasma membrane (PM) (Figure 5A). However, in addition to the PM, mCherry-VAMP721 was also localized in an intracellular compartment (indicated by white arrows). To explore whether the intracellular trafficking of VAMP721 is associated with the endocytic pathway, we administered BFA treatment and observed that mCherry-VAMP721 shifted to the BFA compartments, co-localizing with PIN1-GFP (Figure 5B, white arrows). In the *syp132* mutant, endogenous PIN1 and DsRed-VAMP722 co-localized in an intracellular aggregate (Figure 5C, white arrows). Both *Arabidopsis* VAMP721 and VAMP722 are recognized as functionally redundant v-SNAREs. Notably, single mutants of *vamp721* and *vamp722* exhibited no developmental defects (Figure 5D), and PIN1-GFP localization remained normal (Figure 5E). In contrast, the *vamp721 vamp722* double mutant displayed severe root developmental defects (Figure 5D), with PIN1-GFP primarily localized in intracellular compartments (Figure 5E, white arrows), indicating that PIN1 trafficking is compromised in this double mutant. Collectively, these findings suggest that the PM-localized t-SNARE SYP132 collaborates with the endosome-localized v-SNAREs VAMP721 and VAMP722 to regulate the cycling of PIN1 between the endosome and the PM.

### 2.5. SYP132 Influences Root Growth by Mediating the RGF-PLT Signaling Pathway

**Figure 6:**
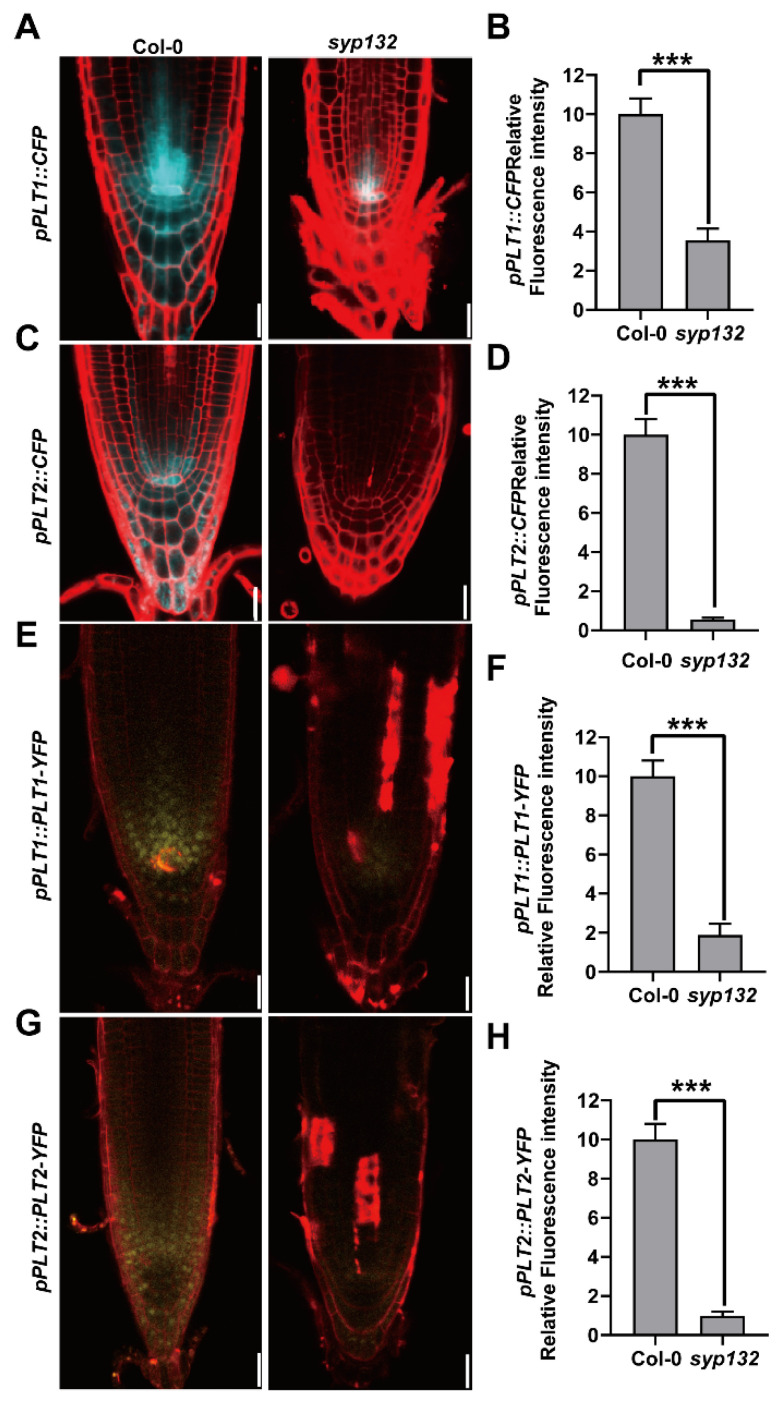
Auxin-PLT pathway is affected in *syp132*. Alteration of PLT1 and PTL2 expression in *syp132*. Expression of *pPLT1::CFP* (**A**,**B**), *pPLT2::CFP* (**C**,**D**), *pPLT1::PLT1-YFP* (**E**,**F**), and *pPLT2::PLT2-YFP* (**G**,**H**) in root tips of 7-day-old Col-0 and *syp132*. Statistics of the relative fluorescence intensities of *pPLT1::CFP* (**B**) shown representatively in (**A**), of *pPLT2::CFP* (**D**) in (**C**), of *pPLT1::PLT1-YFP* (**F**) in E, and of *pPLT2::PLT2-YFP* (**H**) in (**G**). Bars, 20 µm. Values are means ± SD (*n* ≥ 60) from three biological replications. *** *p* < 0.001; Student’s *t*-test.

PLTs and SHR/SCR are TFs participating in the specification and maintenance of the root SCN [4,8,57,58,59]. To examine whether these TFs were perturbed in *syp132*, we introduced *PLT1_pro_:CFP*, *PLT2_pro_:CFP*, *PLT1_pro_:PLT1-YFP*, *PLT2_pro_:PLT2-YFP*, *SCR_pro_:SCR-GFP*, and *SHR_pro_:SHR-GFP* into *syp132* by crossing. The roots of *syp132* exhibited markedly reduced CFP fluorescence, which can be attributed to diminished promoter activities of PLT1 and PLT2 (Figure 6A–D). Correspondingly, a significant decrease in protein accumulation of PLT1-YFP and PLT2-YFP was observed in the roots of *syp132* (Figure 6E–H). We conducted a further analysis of the expression levels of *PLT1* and *PLT2* in the roots of *syp132* by employing RT-qPCR. As anticipated, the expression levels of *PLT1* and *PLT2* were significantly reduced in the roots of *syp132* (Figure S6). However, *SCR_pro_::SCR-GFP* and *SHR_pro_:SHR-GFP* signals in *syp132* were just a little weaker than those in Col-0, indicating the SCR/SHR pathway was not significantly affected in *syp132* (Figure S7).

**Figure 7:**
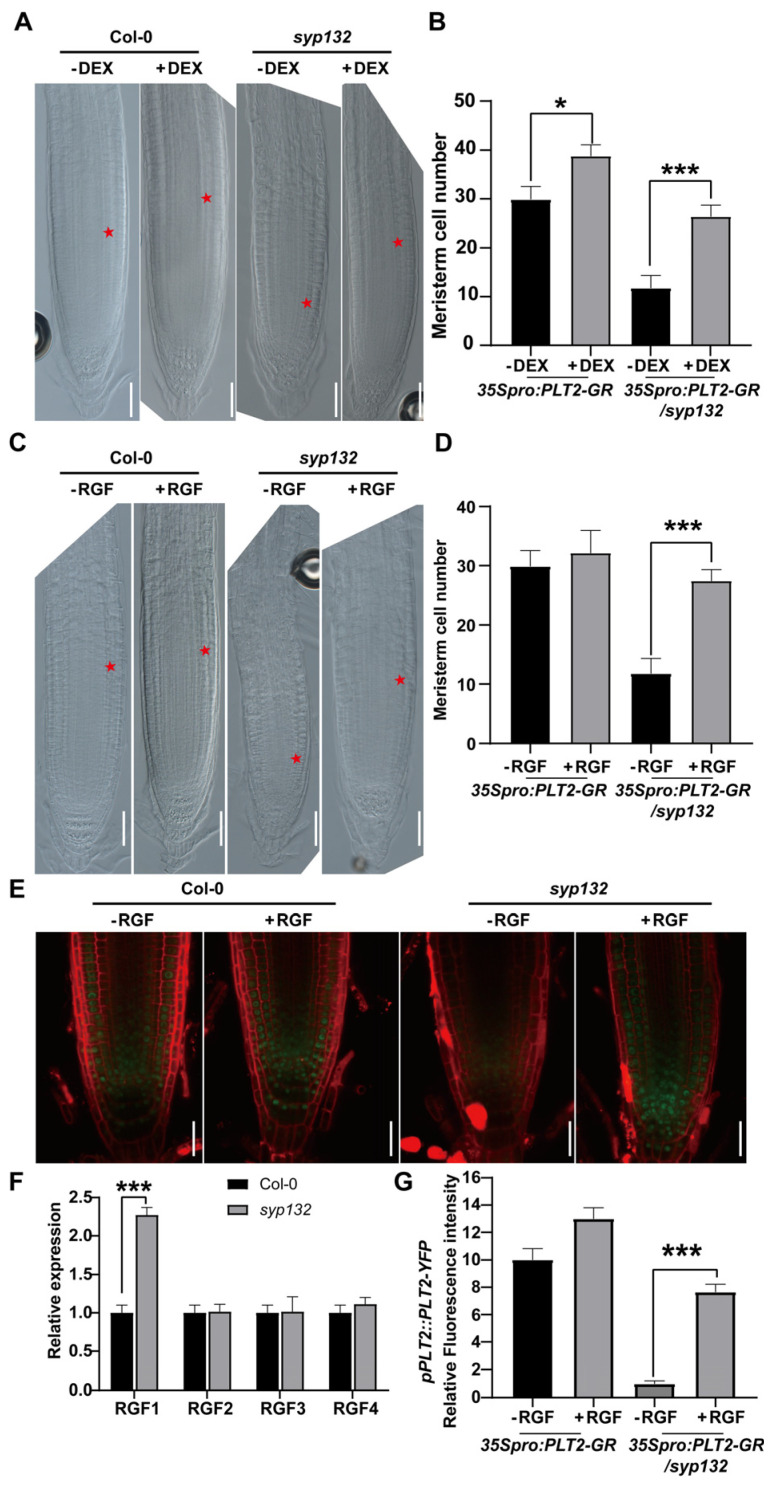
RGF1 peptide partially rescued *syp132* root defects. (**A**) Inducible expression of *35S_pro_:PLT2-GR* under 50 µM DEX treatment. Bars, 20 µm. (**B**) Statistics of meristem cell number of the roots in (**A**). *n* = 30 roots; * *p* < 0.05; *** *p* < 0.001. (**C**) Root growth assay. 5-day-old seedlings were transferred into 1/2 MS liquid medium with or without 100 nM of synthesized RGF1 peptide for overnight before image acquired. Red asterisk indicates meristem size. Bars, 50 µm. (**D**) Statistics of meristem cell number of the roots in (**C**). *n* = 30 roots. (**E**) PLT2-YFP recovery assay. Seedlings were grown on medium with or without 100 nM synthesized RGF1 peptide for 5 days before confocal microscope analysis. (**F**) Three independent experiments per sample, three repeats per experiment. The transcription levels of *RGF* precursor genes were normalized to the ACTIN7 expression. (**G**) Quantification of PLT2-YFP fluorescence intensity in (**D**). *n* ≥ 15 roots; *** *p* < 0.001.

PLTs act downstream of auxin to regulate root SCN maintenance [4,5]. To clarify whether reduced *PLT1* and *PLT2* expression is indeed related to the *syp132-1* phenotype, we generated a dexamethasone (DEX)-inducible *PLT2* overexpression line, *35S_pro_:PLT2-GR*, and crossed it into *atsyp132-1.* Forty-eight hours after DEX induction, the cortex meristem cell number increased 17.49% in *35S_pro_:PLT2-GR*, while, in *35S_pro_:PLT2-GR/syp132-1*, the cortex meristem cell number increased 53.88% but was not recovered to that in Col-0 (Figure 7A,B). This result suggests that SYP132-regulating SCN maintenance is linked to the auxin-PLT pathway.

It was shown that RGFs, the peptide hormone defining the expression of PLTs, are involved in root apical meristem maintenance by directly regulating *PLT* gene expression [7]. RGFs belong to secreted peptides [7,11,60]. Because of SYP132 effects on both membrane secretion and regulation of *PLT* expression, we first analyzed the expression of RGF genes in *syp132*. As shown in Figure 7F, only the expression of the RGF1 precursor gene was altered in *syp132*; the RGF2/3/4 precursor genes did not change. Then, we investigated the *syp132* response to RGF1. Under treatment of synthesized RGF1 peptide, the meristem cell size in the *syp132* mutant was partially recovered (Figure 7C,D). All these results suggest that the RGF pathway was defective in *syp132*. To investigate whether the RGF pathway is related to the PLT pathway, we examined the *PLT2_pro_:PLT2-YFP* response to RGF1. Under RGF1 application, PLT2-YFP accumulation in *syp132* PR was partially recovered (Figure 7E,G). Taken together, we propose that SYP132 affects the PLT pathway in a RGF1-dependent manner.

## 3. Discussion

### 3.1. SYP132 Is Required for QC and Root CSC Identity Maintenance

It is well known that SNAREs play a key role in mediating membrane fusion during vesicle trafficking [18], but the mechanisms underlying SNARE-dependent regulation on SCN maintenance and root development remain elusive. In this study, we demonstrated that PM-localized t-SNARE SYP132 plays an essential role in the maintenance of SCN and RAM activities and root growth and development via regulating intracellular trafficking of auxin efflux carriers, PINs. Due to polar localization and functional relevance for auxin, PIN1 has long been the reference hub protein to decipher auxin flux [61]. Previous studies demonstrated that PIN proteins are internalized via clathrin-mediated endocytosis [51,53], and the GNL1/GNOM-mediated early secretory pathway selectively regulates PIN1 basal polarity establishment in a manner essential for plant development [50,62]. Nevertheless, the molecular mechanism by which PIN1 interacts with the plasma membrane has yet to be thoroughly elucidated.

The SCN maintenance is regulated by many factors, including phytohormones [46,63], TFs [6], peptides [7], miRNA [64], ROS, and glutathione redox [65] status. It is reported that KNOLLE and VAMP721/722 function on root development by interacting with each other on the cell plate during cytokenisis [35,66,67]. Our study revealed that SYP132 interacts with VAMP721/722 to mediate PIN1 targeting to the PM during interphase. First, AtSYP132 is co-localized with VAMP721 on the PM (Figure 5A). Second, SYP132 defects blocked PIN1 targeting to the PM and led to PIN1 ectopic accumulation on the endosome, consequently resulting in the impairment of PIN1 retrograde transport (Figure 4). Third, PIN1 is co-localized with VAMP721/722 on intracellular aggregates, either under BFA treatment or in the *syp132* mutant (Figure 5B,C). Fourth, PIN1 is also accumulated inside the cells rather than on the PM in the *vamp721 vamp722* double mutant (Figure 5E). Taken together, all these data suggest that SYP132 and VAMP721/722 cooperate and are essential for PIN1 targeting to the PM.

### 3.2. SYP132-Mediated Vesicle Trafficking Pathway Is Related to Auxin-PLT Pathway and RGF-PLT Pathway on SCN Maintenance

Because of the perturbed distribution of PINs in the root tip of *syp132*, the SYP132-regulating root growth is related to auxin. Due to the essential role of the auxin-PLT pathway in root development [4,6], we firstly highlight the relation between the SYP132-regulating pathway and auxin-PLT pathway in SCN maintenance. On one hand, *SYP132* deficiency inhibited the transcriptional expression of auxin-response TFs, *PLT1*, and *PLT2* (Figure 6 and Figure 7), probably through the disturbed auxin distribution (Figure 3 and Figure S3). On the other hand, overexpression of *PLT2* could partially rescue RAM defects in *syp132* roots (Figure 6). All these results suggest that the SYP132-regulating pathway works upstream of the auxin-PLT pathway on SCN maintenance and root growth and development. The findings also raise clues that knockdown of *SYP132* might disturb some other factors that directly affect PLT expression. As a secreted root meristem growth factor, RGFs control SCN maintenance by directly modulating the *PLT* expression pattern. In *rgf1rgf2rgf3*, *tpst-1*, and *aqc1-1* mutants, *PIN* expression and auxin signaling were both affected. Since the similar peptide hormone CLV3 was secreted to the extracellular space through the secretory pathway [68] and the RFG precursors are localized on the ER and are targeted to the secretory pathway [11], we were encouraged to explore whether RGFs are involved in SYP132-mediated root SCN maintenance. qRT-PCR indicated that expression of the *RGF1* precursor was increased in *syp132* (Figure 7A). After synthesized RGF1 application, PLT2-YFP protein accumulation was partially recovered, and the defective root SCN phenotype in *syp132* was partially rescued in both RAM size and RAM cell number (Figure 7). The rescue of the *syp132* phenotypes by exogenous RGF1 suggests that secreted mature RGFs may be decreased in *syp132*. All our findings suggest that SPY132-controlled root SCN maintenance is closely related to the auxin-PLT and RGF-PLT pathways, while an interesting topic is how SYP132 regulates RGF secretion. The fact that RGF partially rescued *syp132* root defects suggests the existence of regulation from other factors or pathway(s). *SYP132* plays a significant role in the positive regulation of plant root growth and development. Our study has enhanced the understanding of *SYP132* function and established a foundation for its potential applications in crop improvement. Future research should investigate its molecular mechanisms and application potential to further advance the fields of plant root biology and agricultural science. By modulating *SYP132* expression, it may be possible to develop crop varieties with improved root systems and increased stress tolerance, thereby addressing the challenges posed by global food security and climate change.

## 4. Materials and Methods

### 4.1. Plant Materials and Growth Conditions

*A. thaliana* ecotype Columbia-0 (Col-0) was used as wild-type plants. The marker lines *CYCB1;1:GUS* [69], *PIN3_pro_:PIN3-GFP*, *PIN4_pro_:PIN4-GFP* and *PIN7_pro_:PIN7-GFP* [49], *PIN4_pro_:GUS* [70], *PIN7_pro_:GUS* [71], *QC25:GUS* [13], *QC184:GUS* [13], *WOX5_pro_:GFP* [49], *PLT1_pro_:CFP* [6], *PLT2_pro_:CFP* [6], *PLT1_pro_::PLT1-YFP* [6], *PLT2_pro_::PLT2-YFP* [6], *SHR_pro_::SHR-GFP* [59], *SCR_pro_::SCR-GFP* [72], and *35S_pro_:PLT2-GR* [6], respectively, were employed. Seeds were surface-sterilized with 75% ethanol for 10 min and were rinsed in 100% ethanol once. Ethanol was discarded, and the seeds were dried in the fuming hood. Ethanol was discarded, and the seeds were dried in the fuming hood. Sterilized seeds were sown on 1/2 MS media (1% sucrose, 0.8% agar, pH = 5.7). Seeds were stratified at 4 °C for two days in the darkness. Plants were grown under a 16h:8h/light:dark cycle at 22 °C.

### 4.2. Plasmid Construction and Plant Transformation

For complementation of *syp132*, a 5036 bp DNA fragment, including the 2575 bp *AtSYP132* genomic coding region and the 1825 bp upstream and 636bp downstream sequence, was cloned into binary vector pMDC99 between the PacI and AscI sites; primers were wh359 and wh360. Agrobacterium GV3101 transformed with the plasmid *SYP132_pro_:gSYP132/pMDC99* was used for floral dip [73] using heterozygous *syp132-1^+/−^* plants. To generate the *SYP132_pro_:GUS* transgenic plants, the *SYP132* promoter sequence was first cloned into the pDONR207 ENTRY vector by a BP recombination reaction using primers *wh328* and *wh329* and then transferred into the destination vector pMDC162 by an LR recombination reaction (Invitrogen, Carlsbad, CA, USA). As for *SYP132* fused with fluorescent protein tag vectors, firstly, the GFP tag in pMDC43 was replaced by sGFP and dsRED, giving rise to the two new vectors pMDC43-sGFP and pMDC43-dsRED. Due to the trans-membrane domain in the C-terminal of the SYP132 protein, GFP and dsRED were fused in the N-terminal of the SYP132 protein. The *SYP132* promoter fragment with 5′-MssI and 3′-KpnI was amplified from the genome of a wild-type (Col-0) seedling with the two primers wh432/wh433 and replaced the original CaMV35S promoter of *pMDC43-GFP* and *pMDC43-dsRED*, generating the two new vectors *pMDC43-SYP132pro:GFP* and *pMDC43-SYP132pro:dsRED.* The fragment of the SYP132 genomic sequence with 5′-AscI and 3′-PacI was amplified with the primers wh362/wh363 from the genome of the Col-0 seedling and cloned into the AscI and PacI sites of the *pMDC43-AtSYP132pro:GFP* and *pMDC43-SYP132pro:dsRED* vectors, generating *pMDC43-SYP132pro:GFP-gSYP132* and *pMDC43-SYP132pro:dsRED-gSYP132*, respectively. The VAMP722 promoter with 5′-MssI and 3′-KpnI was amplified from the genome of Col-0 with the two primers wh560/wh561 to replace the CaMV35S promoter of *pMDC43-dsRED*, having the *pMDC43-VAMP722pro:dsRED* vector. The VAMP722 genomic DNA sequence with 5′-AscI and 3′-PacI was amplified from the genome of Col-0 with the two primers wh565/wh566 and inserted into the AscI and PacI sites of *pMDC43-VAMP722pro:dsRED*, generating the vector *pMDC43-VAMP722pro:dsRED-gVAMP722. Agrobacterium tumefaciens* GV3101 was used to transform heterozygous *atsyp132-1* via the floral dip method as reported by Clough and Bent [73].

### 4.3. Phenotype Analysis

Six-day-old seedlings were scanned, and primer root lengths were measured using ImageJ (Version: 1.53t, National Institutes of Health, USA). For Nomarski imaging of roots, seedlings were fixed and mounted in chloral hydrate solution [65] (chloral hydrate:water:glycerol = 8:3:1). Images were recorded with an AxioVision microscope (Carl Zeiss Microimaging; Axioscope 2, Oberkochen, Germany) [65]. RAM size was also measured via ImageJ (Version: 1.53t, National Institutes of Health, USA); the length between the QC and the first elongating cell in the cortex cell file was defined as the RAM size [65,74]. For propidium iodide (PI) staining, 6-day-old seedlings were incubated in PI (10 μg mL^−1^) for 1 min and then directly viewed with confocal microscopy after mounting with water. For mPS-PI staining, seedlings were vacuumed for 2 min in 2% PFA in 1 × MTSB (7.5 g PIPES; 0.95 g EGTA; 0.66 g MgSO_4_.7H_2_O; 2.5 g KOH; H_2_O up to 500 mL pH = 7.0). The vacuum process was performed twice. After that, samples were incubated in fixative for an additional 40 min. Then, seedlings were rinsed in H_2_O for 5 min. After two times washing step, the seedlings were treated in 1% periodic acid for 20 min. The seedlings were rinsed two times with water and incubated with 100 μg mL^−1^ PI for 10 min. After two times washing, the seedlings were mounted in mounting solution. During the incubation, the samples must be protected from light. For Lugol staining, prior to microscope inspection, *Arabidopsis* seedlings were stained with 1% Lugol reagent (1% iodine; 2% potassium iodine) for 10 min. Then, the seedlings were mounted on microscope slides in mounting solution.

### 4.4. Histochemical Analysis

For GUS staining, we adapted the method as described [75]. Different stage seedlings, as indicated, were fixed in cold 90% acetone for 15 min on ice; then, the seedlings were rinsed with GUS staining buffer (100 mM phosphate buffer, pH 7.0, 10 mM EDTA, 50 mg L^−1^ 5-bromo-4-chloro-3-indolyl-β-glucuronic acid, 0.1% Triton X-100, 5 mM K_3_Fe [CN]_6_, and 0.5 mM K_4_Fe [CN]_6_) without X-Glucuronide. Next, the plants were incubated in GUS staining buffer containing 1 × X-Glucuronide (50 mg mL^−1^ in DMSO diluted 1:100) and under vacuum for 3 min, which was repeated three times. Then, the samples were subsequently incubated at 37 °C for a period ranging from 20 min to four h. After staining, the samples were washed several times with 95% ethanol and were then incubated in water and mounted in chloral hydrate solution (chloral hydrate:water:glycerol = 8:3:1). An AxioVision microscope (Carl Zeiss Microimaging; Axioscope 2, Oberkochen, Germany) was employed for high-power magnification of the stained samples. For low-magnification imaging, we utilized an AxioVision stereo microscope (Carl Zeiss Microimaging; Axioscope 2, Oberkochen, Germany) paired with an AxioCam MRc digital camera (Carl Zeiss Microimaging; Axioscope 2, Oberkochen, Germany), which typically provides 10× magnification through the eyepiece and 20× magnification with the objective lens.

### 4.5. RNA Isolation, RT-PCR, and qRT-PCR

Total RNA was extracted from different organs, including roots, leaves, shoots, flowers, and siliques and from seven-day-old Col-0 or *syp132* mutant true leaves using the RNeasy plant mini kit (Qiagen 74903, Hilden, Germany) and digested with TURBO DNase (Ambion 2239, Austin, TX, USA). The extracted RNA was reverse transcribed into cDNA using the High-Capacity cDNA reverse transcription kit (Applied Biosystems 4374966, Waltham, MA, USA). RT-PCR was used to check *SYP132* expression in the T-DNA insertion mutants. All primer pairs used are presented in Table S1. Gene expression was assessed by quantitative RT-PCR (qRT-PCR). Total RNA from 2 mm root tips of 10-day-old seedlings was extracted using the innuPREP Plant RNA kit (Analytic, Jena, Germany). Before cDNA synthesis, the RNA sample was incubated with 1 μL DNase I at 37 °C for 30 min (Thermo Scientific EN0521, Waltham, MA, USA), followed with 1 μL 50 mM EDTA at 65 °C for 10 min to remove trace amounts of DNA. First strand cDNA was synthesized by the RevertAid H Minus First Strand cDNA Synthesis Kit (Thermo Scientific K1631, Waltham, MA, USA). A total of 1 μg total RNA and 1 μL oligo(dT)_18_ primer, 4 μL 5 × Reaction Buffer, 1 μL RiboLock RNase Inhibitor (20 u μL^−1^), 2 μL 10 mM dNTP Mix, and 1 μL Revert Aid H Minus M-MuLV Reverse Transcriptase (200 u μL^−1^) were added into the 1.5 mL nuclease-free Eppendorf tube. Nuclease-free water was added to a total volume of 20 μL reaction mix. The mixture was incubated at 42 °C for 60 min. The reaction was terminated by heating at 70 °C for 5 min. qRT-PCR was performed with the Maxima SYBR Green qPCR master mix (Thermo Scientific A25741, Waltham, MA, USA) on an ABI 7300 real-time PCR system (Thermo Scientific™ Applied Biosystems (ABI) 7300 PULAS real-time PCR instrument, Waltham, MA, USA). The thermocycle program was as follows: 10 min of preincubation at 95 °C, followed by 35 cycles of 15 s at 95 °C, 15 s at 56 °C, and 30 s at 72 °C. ACTIN was used as a reference gene. The relative gene expression was analyzed via the ∆-∆ cycle threshold method. Three biological replicates and three technical replicates were used to evaluate gene expression.

### 4.6. SYP132 Antibody Generation and Immunoblot Assay

To generate the *SYP132*-specific antibody, we used two primers, PSYP132 antibody synthesis fwd and PSYP132 antibody synthesis rev, to clone SYP132 cDNA, corresponding to amino acid from 5 to 200, into NdeI/SalI of vector pET-28(a) (Novagen 11905ES03, Madison, WI, USA). After expression in *E. coli* strain BL21 golden star, the recombinant protein containing the His6 tag was affinity purified according to the Qiagen manual (Qiagen, Hilden, Germany) and checked by SDS-polyacrylamide gel electrophoresis. The antigen peptide included in PAGE-slice was used to immunize a rabbit (Eurogentec 203681, Seraing, Belgium). The polyclonal antiserum was affinity purified against the recombinant SYP132 peptide. To extract the protein, 10-day-old seedlings (0.3 g) were homogenized on ice in 1 mL of extraction buffer (50 mM HEPES-KOH, pH = 6.5, 10 mM potassium acetate, 100 mM sodium chloride, 5 mM EDTA, and 0.4 M sucrose) with protease inhibitor mixture (Sigma 798681, St. Louis and Burlington, MA, USA). The homogenate was centrifuged at 500× *g* for 5 min. The debris were discarded. The supernatant was transferred into a new Eppendorf tube and centrifuged at 10,000× *g* for 15 min to generate a membrane pellet. To solubilize the membrane proteins, the pellet was resuspended in 750 μL of extraction buffer containing 1% (*v*/*v*) Triton X-100 and protease inhibitor mixture and incubated at 4 °C for 2 h (rotation). The mixture was recentrifuged at 10,000× *g* for 15 min. After that, the insoluble material was discarded. For immunoblot analysis, the supernatant was boiled with 5 × SDS loading buffer for 5 min. The supernatant was collected and subjected to immunoblot using anti-SYP32 antibody (1:500).

### 4.7. BFA Treatment

Seven-day-old seedlings were incubated in half-strength MS liquid medium containing 50 µM Brefeldin A (BFA)and 50 µM cycloheximide (CHX) or dimethyl sulfoxide (DMSO) and 50 µM CHX as a control for 45 min, respectively. For the washing-out experiment, seedlings were first treated with 50 µM BFA and 50 µM CHX for 90 min and then washed twice with 1/2 MS liquid medium and incubated in 1/2 MS liquid medium for 90 min for recovery. For the evaluation of *syp132-1* oversensitivity to BFA, the PIN1-GFP BFA compartments and PM PIN1-GFP fluorescence intensity were measured via Imaris 7.0 × 64 software (Bitplane AG, Zurich, Switzerland). Then, the ratio of PIN1-BFA compartments fluorescence intensity and PM fluorescence intensity was calculated.

### 4.8. Immunolocalization

Seven-day-old seedlings were fixed with 4% paraformaldehyde in PBS (pH 7.3) and used for whole-mount in situ immunolocalization in root as previously described [48]. The primary antibodies were diluted at the following concentrations: anti-PIN1 mouse, 1:20; anti-PIN1 rabbit, 1:500; anti-GFP mouse (Roche 11814460001, Basel, Switzerland), 1:500; anti-RFP mouse (Roche 11814460001, Basel, Switzerland), 1:1000. For the secondary antibodies, Alexa Fluor 488 goat anti-mouse IgG (Invitrogen K0038M, Carlsbad, CA, USA), 1:500; Alexa Fluor 555 goat anti-mouse IgG (Invitrogen 0102354, Carlsbad, CA, USA), 1:500; Alexa Fluor 488 goat anti-rabbit IgG (Invitrogen K0038M, Carlsbad, CA, USA), 1:500; and Alexa Fluor 555 goat anti-rabbit IgG (Invitrogen 0102354, Carlsbad, CA, USA), 1:500 were used. Samples were mounted with Slow Fade Antifade kit (Life Technologies S2828, Carlsbad, CA, USA).

### 4.9. Confocal Microscopy

GFP, YFP, CFP, and PI fluorescence were photographed under a Zeiss LSM 510 microscope (Carl Zeiss Microimaging, Oberkochen, Germany). For GFP/eGFP, Venus, CFP, and PI, the 488, 514, 458, and 561 nm lines of the laser were used for excitation, and emission was detected at 505–530, 530–600, 465–520, and 590–635 nm, respectively. Zen2009 (Carl Zeiss Microimaging, Oberkochen, Germany) was used to process and extract the images. For analyzing RoGFP2, wavelengths of 405 and 488 nm were used for excitation, respectively, and emission was detected at 505–530 nm.

### 4.10. RGF1 Synthesis and Plant Treatment with RGF1

RGF1 was synthesized according to the amino acid sequence described by Matsuzaki et al. [12]. For the root growth assay, 5-day-old seedlings were transferred into 1/2 MS liquid medium containing 100 nM of synthesized RGF1 peptide for overnight incubation before images were acquired. Root length was measured using ImageJ software (Version: 1.53t, National Institutes of Health, USA). For the PLT2-YFP recovery assay, seedlings were grown on 1/2MS solid medium with or without 100 nM synthesized RGF1 for 5 days before confocal microscope analysis [76].

## Data Availability

Data are contained within the article and Supplementary Materials.

## Supplementary Materials

The following supporting information can be downloaded at: https://www.mdpi.com/article/10.3390/ijms26052123/s1.
