# Supplementary material for: SYNTAXIN OF PLANTS132 Regulates Root Meristem Activity and Stem Cell Niche Maintenance via RGF-PLT Pathways
Source: Int J Mol Sci. 2025 Feb 27;26(5):2123. doi: 10.3390/ijms26052123 (PMC11900091; doi:10.3390/ijms26052123)
Supplement: Supplementary file 1 [file ijms-26-02123-s001.zip › ijms-3475337-supplementary .pdf]

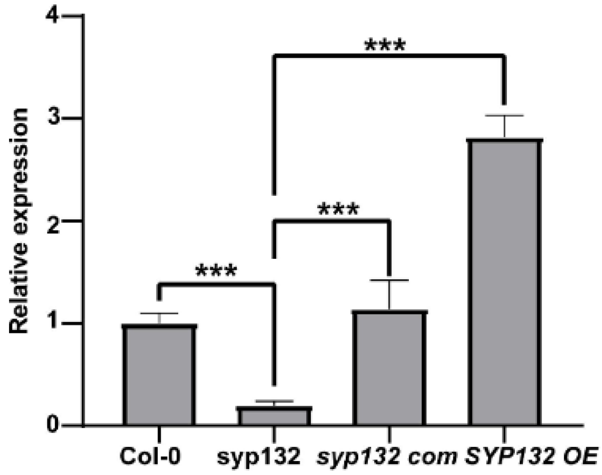

Supplemental Figure S1.RT-qPCR in Col-0, *syp132*, *syp132 com* and *SYP132 OE*. Statistics of RT-qPCR analysis of the relative expression levels of SYP132 in seven-day-old seedlings of the indicated genotypes. Data are presented from three independent experiments performed with four technical replicates per experiment. The error bars in this study are standard variance (SD). Data are presented from three independent experiments performed with three technical replicates per experiment. Asterisks represent highly significant differences. (**, P<0.01, Student's t test).

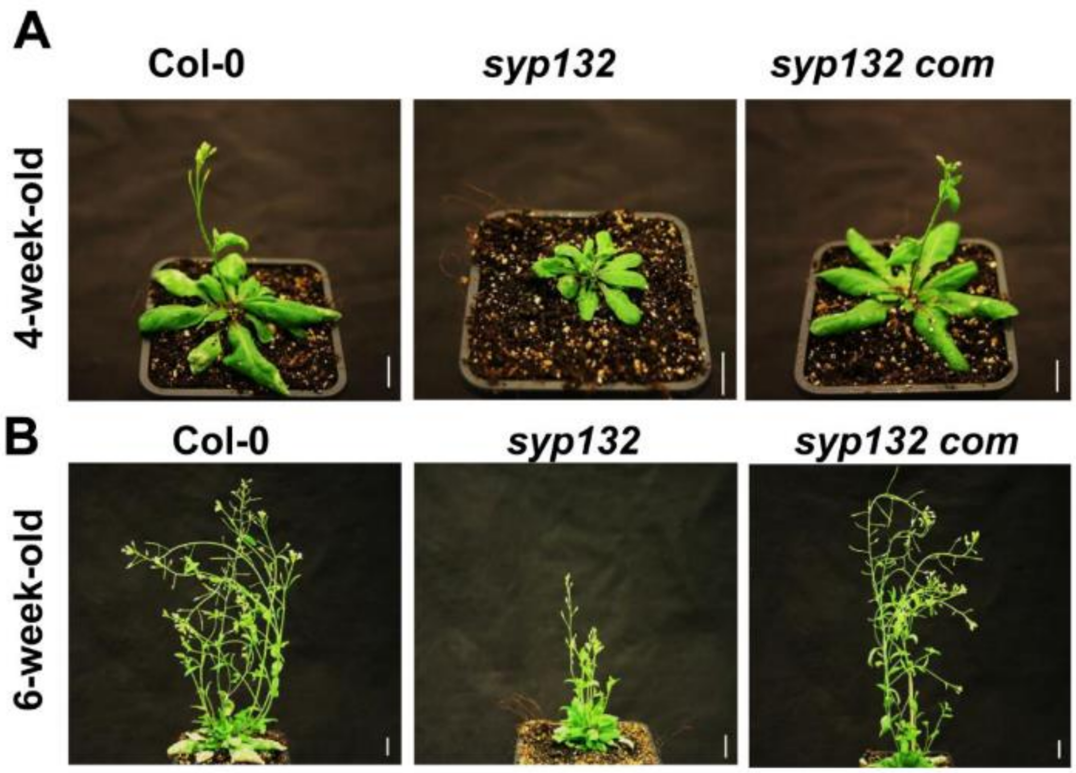

Supplemental Figure S2.*syp132* exhibits development defects.

A.4-week-old plant of WT, *syp132* and complemented plants. Bars =10 mm. **B.** 6-week-old plant of WT, *syp132* and complemented plants. Bars=10 mm.

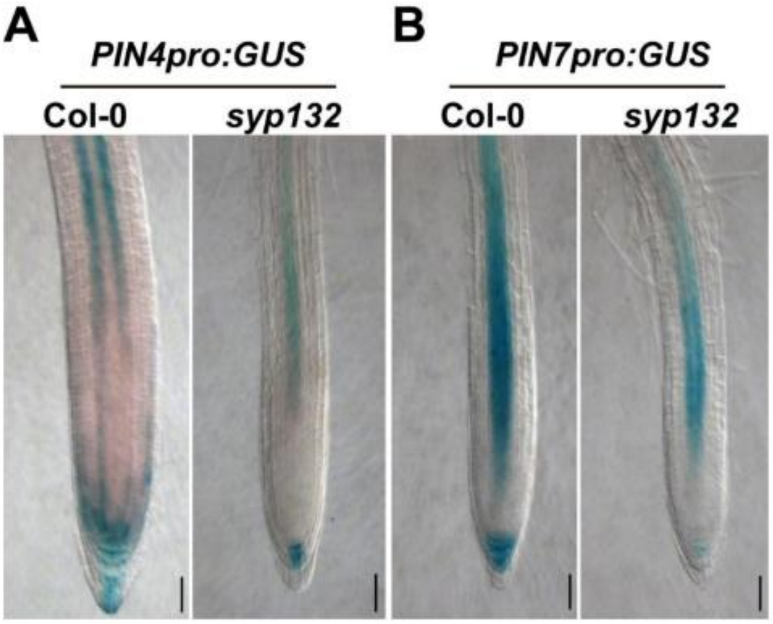

Supplemental Figure S3.The expression of PIN4 and PIN7 are altered in *syp132*.

PIN4pro:GUS shows decreased expression of PIN4 in *syp132* compare with Col-0 . Bars = 20 μm. **B.** *PIN7pro:GUS* shows decreased expression of PIN7 in *syp132* compare with Col-0. Bars = 20 μm.

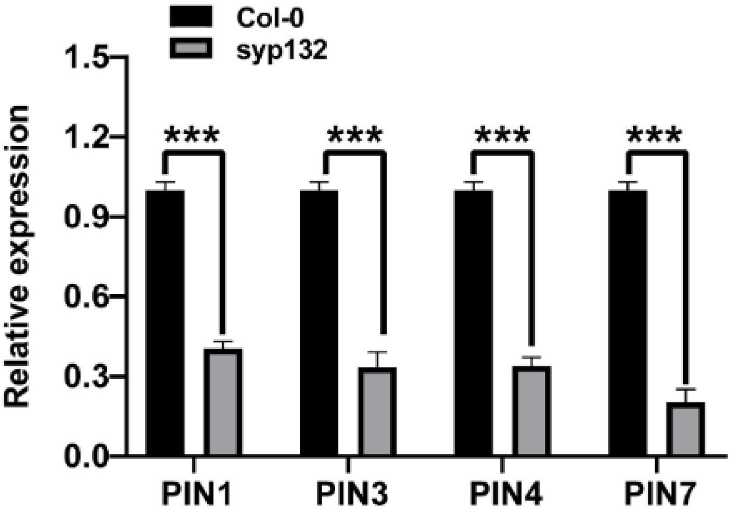

Supplemental Figure S4. Expression of *PIN1*, *PIN3* ,*PIN4* and *PIN7* in Col-0 and *syp132* roots. **Statistics of RT-qPCR analysis of PINs expression levels in roots of *syp132* seedlings. Data are presented from three independent experiments performed with three technical replicates per experiment. *** *P* <0.001; Student's t-test.**

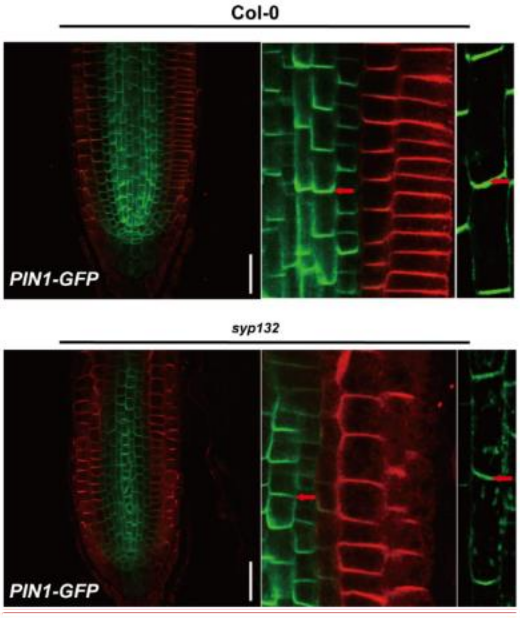

Supplemental Figure S5. PIN1 is affected in *syp132* . Alteration of PIN1 expression in *syp132* . Bars, 20 μm.

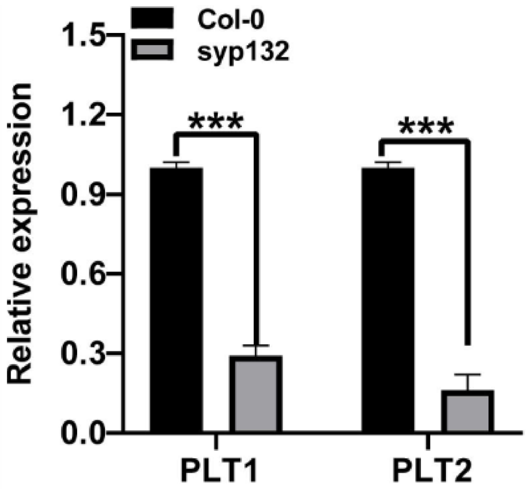

Supplemental Figure S6. Expression of *PLT 1* and *PLT2* in Col-0 and *syp132* roots.
Statistics of RT-qPCR analysis of PLTs expression levels in roots of *syp132* seedlings. Data are presented from three independent experiments performed with three technical replicates per experiment. ****P*<0.001; Student's t-test.

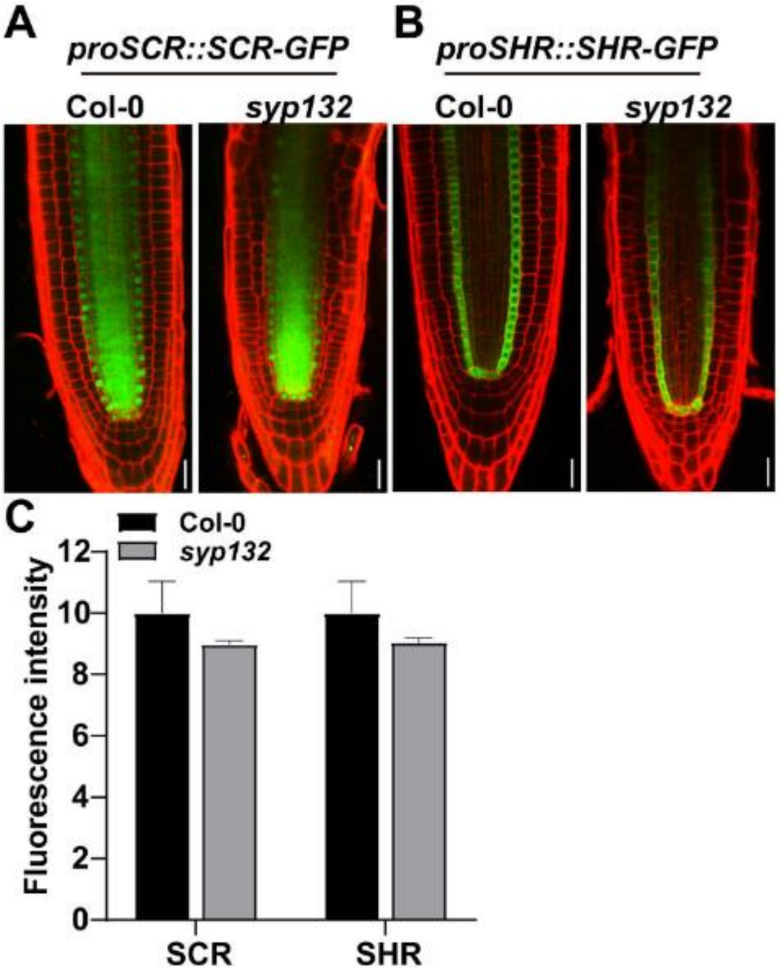

Supplemental Figure S7. Expression profile of *SHR* and *SCR* in Col-0 and *syp132* mutant.

A. Fluorescence analysis of 7-day-old Col-0 and *syp132* mutant seedlings expressing *proSCR::SCR-GFP* and *proSHR::SHR-GFP* (**B**). Scale bars: 500μm. Data are presented from three independent experiments performed with four technical replicates per experiment. **C.** Statistics of RT-qPCR analysis of *SCR* and *SHR* expression levels in roots of *syp132* seedlings. Data are presented from four independent experiments performed with three technical replicates per experiment.

Supplemental tables

Table S1. Primers used in vector construction and RT-qPCR

**Oligonucleotide names**	**Sequence (from 5' to 3')**
wh328	AAAAAGCAGGCTAAATTCACACGTTGTTTCAT
wh329	AGAAAGCTGGGTACTTATCATACTGTTTGTC
wh359	CCCCTTAATTAAGCATGCGCTTGACCGACCAC
wh360	AAAAGGCGCGCCTGGTTGTCTTCTTCGAAGC
wh362	CCCCTTAATTAAGGATTCAGTGCAGCGGATG
wh363	AAAAGGCGCGCCCATTTGAGGTCACATAGACG
wh432	GTTTAAACGCATGCGCTTGACCGACCAC
wh433	GGTACCCGTGTAGAGCAAAAAGATAG
wh423	CAACAGTGGTCGGCATTCATGA
wh424	TGCACATGAGTCGCACATGATG
wh425	ATTTTGCCGATTTCGGAAC
wh426	CCCCCGTCCATTAAGAATTAAG
wh427	TATCAACCAAAGCTACCACGG
wh429	GATCTACAGTTTCGTCGCTCG
wh430	CTTCTCACCACGGTCAAGAAC
wh560	GGGGTTTAAACACTGAGTGATCCTGAGGTCG
wh561	GGGGGTACCTTTTTTTACTCAAAAACGACGCG
wh565	AAAAAGCAGGCTGGATGGCGCAACAATCGTTG
wh566	AGAAAGCTGGGTGCCCTTAATGAACTAACCGG
AtSYP132 antibody synthesis fwd	CCCCCATATGCTGAAGGGTTCGTTTGAGCTTC

AtSYP132 antibody synthesis rev	AAAAGTCGACTACCTGTCCTCTTCCTTGCTCC
PAtSYP132 qRT-PCR fwd	GCGTCTATTGTCTTCGCACT
PAtSYP132 qRT-PCR rev	CCTTGATCTCCACCTTGTTG
RGF1qPCR-F	GGTGTCCATAAGGGTTATTTGC
RGF1qPCR-R	TGCTCCAAGACCTTCACTATTT
RGF2qPCR-F	GCTTTCTGTGTTTGTTGATCCT
RGF2qPCR-R	TCAACACTTAGCACTTCATCCT
RGF3qPCR-F	TGAAGATGGGAATCAACAGTCA
RGF3qPCR-R	ATCAGCAGTGTAAGCTACAAGT
RGF4qPCR-F	GAGGAAAGTAAGGTTTGGGAGA
RGF4qPCR-R	GTAGATGGTTCAAGTGTCGGTA
